# The patient/physician relationship in a post-*Roe* world: a neonatologist viewpoint

**DOI:** 10.1038/s41372-022-01583-3

**Published:** 2022-12-17

**Authors:** Maria E. Barnes-Davis, DonnaMaria E. Cortezzo

**Affiliations:** 1 Division of Neonatal and Pulmonary Biology, Cincinnati Children’s Hospital Medical Center, Cincinnati, OH USA; 2 Department of Pediatrics, University of Cincinnati College of Medicine, Cincinnati, OH USA; 3 Pediatric Neuroimaging Research Consortium, Cincinnati Children’s Hospital Medical Center, Cincinnati, USA OH; 4 Department of Anesthesiology, University of Cincinnati College of Medicine, Cincinnati, OH USA; 5 Division of Pain and Palliative Medicine, Cincinnati Children’s Hospital Medical Center, Cincinnati, OH USA

**Keywords:** Medical ethics, Paediatrics, Health policy, Palliative care

## Abstract

The Supreme Court ruling in *Dobbs v. Jackson Women’s Health Organization* has far-reaching implications that go beyond the practice of obstetrics and gynecology. The ruling and subsequent laws and bills impact many specialties and have implications for healthcare as a whole. The rapidly changing medicolegal landscape has significant bearings on and implications for the fields of neonatology and pediatrics. These rulings have an impact on the patient-physician relationship and a shared decision-making approach to care. Furthermore, there are significant sequelae of forced birth and resuscitation. This review provides a clinically relevant update of the current medicolegal landscape and applications to the practice of neonatology.

## Introduction

The Supreme Court ruling in *Dobbs v. Jackson Women’s Health Organization*, subsequent trigger laws, and proposed state bills reach beyond obstetrics and maternal-fetal medicine (MFM), impacting healthcare as a whole. With the overturn of *Roe* and increasingly restrictive abortion laws, the average distance to travel for abortion-related healthcare will increase by 97 miles [1]. Greater distances are associated with increased burden, delays in care, and decreased service utilization [2–4]. This will adversely impact public health, worsen disparities, and increase maternal and neonatal morbidity and mortality [5–8]. Individuals denied abortions are more likely to experience complications with life-long consequences, including poor mental health, preeclampsia, and death [9].

The ramifications, though, are not limited to obstetrics or fetal status. There are consequences for fertility and reproductive health, oncologic treatment of pregnant individuals, and availability of life-saving medications. Nationally, there have been questions about when physicians can intervene to save a pregnant individual’s life if the intervention may result in abortion; refusal to fill prescriptions for medications used to treat complex diagnoses if the medications can also be used to treat ectopic pregnancies; and criminal charges against a woman who sought care for miscarriage when the prosecutors questioned if the miscarriage was the result of an abortion [5, 10–12]. Recognizing that recent legislation impacts adolescents, the American Academy of Pediatrics (AAP) highlighted the adverse impact and health risks of restricting abortion access [13]. Despite this, politicians disputed that a 10-year-old rape victim could become pregnant, and threatened to prosecute a physician who provided legal abortion care, when the child traveled out of state for abortion [14–16].

This still does not fully encompass the impact of these laws and bills. The American Academy of Hospice and Palliative Medicine expressed concern that the *Dobbs* ruling will interfere with the patient-physician relationship and harm families in need of perinatal palliative care [17]. As neonatologists, we have the privilege of caring for families faced with unexpected fetal and neonatal diagnoses. We counsel pregnant individuals about these diagnoses while providing support and guidance through their journey. For many, these are difficult and personal medical decisions that evolve over time. Recent legislation impacts counseling we provide and care we can offer. These repercussions have not been sufficiently discussed. We present a neonatologist’s perspective on the impact of the current legal climate on neonatal-perinatal medicine and propose ways to navigate through these challenges while providing optimal care.

## Medicolegal landscape

Ceding control over medical decisions regarding abortion to states has led to ancillary legislative activity. Currently, 44 states prohibit abortion at some point in pregnancy and 6 states prohibit abortion when the fetus has a genetic anomaly, some of which may be life-limiting [18, 19]. For example, the Ohio “heartbeat bill” was signed in to law, criminalizing abortion after the presence of a fetal “heartbeat” unless intended to prevent maternal death or severe impairment of a major bodily function [20]. “Heartbeat” is misleading, as the heart is not completely formed and there are many nuances of care this law fails to recognize [21]. Additionally, the language used leaves uncertainty, even to trained physicians, as to what constitutes severe impairment of a major bodily function. There are circumstances where the wishes and well-being of the pregnant individual are placed behind those of the fetus and the individual is expected to risk their health and life for the fetus. Under these new restrictions, complex decisions involving multiple births are also compromised. The well-being of one twin may be jeopardized for that of a twin not expected to survive. Moreover, to preserve the potential life of the fetus, the pregnant individual is effectively mandated to donate their body to sustain this life. Currently, even in death, individuals are not mandated in other circumstances to donate organs or risk their well-being to save a life. Another state bill was passed declaring abortion providers cannot be affiliated with academic institutions and providers must exercise medical judgment to preserve the child’s life after a “failed abortion.” [22] The meaning of these terms is open to interpretation. In addition to suggesting that a previable infant should be resuscitated, the legislation effectively closes clinics, prevents obstetricians from learning important, life-saving procedures, and sets a precedent that medical education and training of physicians can be dictated by those without requisite medical or scientific knowledge [6, 23]. Additional bills designate personhood at conception and imply mandatory resuscitation irrespective of gestational age [24, 25]. While some states claim they will not prosecute physicians providing abortions, other state medical boards with politically appointed non-physician members have threatened to revoke medical licenses [26]. Currently, 18 states have born alive protections requiring practicing “skills” to preserve life and others have proposed similar bills [27].

Collectively, recent legislation establishes a precedent that medical decisions are made by the state, not patients and their physicians. Most lawmakers lack the knowledge or training to understand medical appropriateness and are therefore ill prepared to dictate standards for medical care. For instance, several proposed bills would mandate aggressive treatment for any infant displaying movement, sound, heartbeat, or pulsating umbilical cord. These measures do not independently reflect voluntary movement, effective respirations capable of sustaining life, or effective cardiac output. As a result, this would necessitate futile interventions for previable infants and infants with life-limiting anomalies when they would not be beneficial or if families perceive them as causing suffering. It would significantly alter the end-of-life experience for the family and in certain instances impact their ability to bond and make memories with their baby. It contradicts recommendations from AAP and other medical associations, undermines patient autonomy, and undervalues the patient-physician relationship [28].

The legal implications of these bills and laws may alter options discussed with families. Historically, most states have deferred judgment of viability to physicians [29]. While *State v. Messenger* affirmed parental rights to refuse life-sustaining interventions, *Miller v. HCA* and *Montavola* restricted their role in such decisions [30, 31]. Some feel the Born Alive Infant Protection Act serves to supersede physician judgment and restructure the limit of viability [32]. In reality, it established personhood for infants who breathe, have a heartbeat, or have spontaneous muscle movement. The standards of care, standard practice, and medical treatments are not determined by the presence of personhood. Recent language, though, such as “unborn human being,” creates ambiguity. If there is forced birth for an unborn person, does that necessitate a forced resuscitation? Whether related to periviability or a life-limiting diagnosis, “physicians should not be forced to undertreat or overtreat an infant if, in their best medical judgment, the treatment is not in compliance with the standard of care for that infant.” [33]

## Decision-making

Restricting abortion, redefining personhood, and mandating resuscitation impacts the counseling and care our patients receive. While counseling about life-limiting fetal diagnoses, we discuss appropriate medical options including abortion, pregnancy continuation, and neonatal treatment paths. With increased restrictions, we must remain knowledgeable of state laws to direct patients to appropriate care and prepare families that, given individual time and financial constraints, abortion may not be feasible. This increases the workload for an already over-burdened medical system, decreases the quality of patient care, and worsens inequities in maternal and neonatal-perinatal care. Many women who seek abortions are from marginalized and minoritized ethnic and racial groups and 75% have incomes at or near the poverty line [8].

When parents are excluded from pregnancy and resuscitation decisions, they have higher rates of depression, anxiety, and post-traumatic stress [34]. Perceived stigma regarding abortion, including for fetal anomalies, may contribute to increased trauma, highlighting the importance of navigating these delicate circumstances with non-judgment and compassion [35]. When making decisions about goals of care, parents consider suffering, potential disability, quality-of-life, best interest, maternal mortality, and financial implications within the context of the needs and culture of their family and community [36]. The American College of Obstetrics and Gynecology, AAP, and other societies suggest addressing clinical concerns for the pregnant individual and fetus while providing accurate, unbiased information and guidance to families [37]. Neonatology has also shifted to a shared decision-making process by intertwining family values and physician expertise for the range of ethically permissible treatment options and decisions within the zone of parental discretion [38, 39]. In this context, the physician and family must balance principles of autonomy, beneficence, and nonmaleficence in a manner that promotes best interest, minimizes burden, and incorporates parental views.

Proposed bills mandating pregnancy continuation or resuscitation before viability or with life-limiting diagnoses are harmful and assume that all families desire interventions. It implies elected officials rather than the patient and physician are responsible for making medical decisions. Moreover, the bills are predicated on the assumption that interventions will improve survival, which may be inaccurate. These complex decisions can and should only be made by families and physicians or advanced practitioners who have knowledge and training to understand limitations of interventions, explore family values, and navigate goals of care [40].

## Forced birth and resuscitation

With greater abortion restrictions, whether due to inherent risks with continuation of pregnancy or abortions provided without strict oversight and supervision, maternal and neonatal morbidity and mortality will increase [6]. Pregnant individuals may experience significant health consequences during the prenatal, perinatal, and postpartum periods that can lead to life-long complications and death, impacting the child and the entire family [9, 41]. These barriers will disproportionately impact individuals of racial and ethnic minorities, those with limited access to transportation, adolescents, and individuals with disabilities.

Further highlighting the public health impact, each year there are >15,000 neonatal deaths in the United States and >3% of pregnancies are complicated by a life-limiting diagnosis [42, 43]. Currently, 20–40% of patients continue these pregnancies [44]. Post-*Roe*, whether by parental choice or state mandate, this number will increase. The decision to continue such a pregnancy is incredibly personal and complex. Pregnancy continuation leads to lost income, lost work productivity, lost reproductive potential, and increased risks of medical complications. There is often increased testing, medical visits, hospitalizations, and emotional trauma [45]. These laws will increase the burden of chronic stress and suffering for individuals needing perinatal palliative care [17, 46]. This impacts not only the maternal-infant dyad but siblings and other family, with the potential for transgenerational effects ranging from long-term gene expression changes to effects on brain development [47, 48].

Some suggest hospice as an alternative to abortion for life-limiting or medically complex fetal diagnoses. However, some parents feel this is not in their fetuses’ best interest. Beyond that there remains wide variability in the availability, scope, and services of palliative care programs [49]. Not all diagnoses are conducive to this pathway. Some, such as anencephaly and bilateral renal agenesis, are accepted as life-limiting and neonatal-perinatal palliative care and hospice are routinely offered. The baby may breathe and live for a period of time and some families may experience additional grief and trauma if they believe their baby is suffering [50]. Other diagnoses, such as hypoplastic left heart syndrome, have significant variability around whether comfort measures are offered despite the medical complexity and high mortality risk [51]. Comfort measures are not traditionally offered with other medically complex diagnoses, such as myelomeningocele and cloacal exstrophy. In a time of unprecedented medical resource shortages, the healthcare system is not equipped for downstream effects of more medically complex patients. Concerns about the pediatric workforce’s ability to meet these care needs will increase as there are more children with complex chronic medical needs [52]. Pediatric hospital services, support structures, and homecare services are already experiencing shortages and poor reimbursement for such services, which decreases the likelihood of sustainability [53]. It is unclear how we will continue to provide the highest level of care to these infants.

Irrespective of resources, forced delivery and resuscitation are traumatic for all. It negates physicians’ judgment of what is medically appropriate and families’ right to choose what is in their child’s best interest. We have cared for families with pregnancies complicated by life-limiting conditions who sought abortion but were unable to obtain one. The pregnancy continued and most families elected for comfort care. Many had painful experiences of strangers asking about their baby and the future they anticipate. Some felt guilt and pressure to pursue interventions when they did not feel it was in their child’s best interest. Others valued time with their baby but were traumatized because they were forced to continue their pregnancy and feel their baby suffered. They felt helpless and betrayed, asking what else they could do, how the state could mandate they have a child when lawmakers do not understand what it means for them and their baby, and how they are supposed to give birth knowing their baby is dying. These moments, patients, and conversations stay with us and with the families. Elected officials lack this insight and perspective.

## Continuing to provide complete counseling and care

As neonatologists, we counsel families about all appropriate medical options and support them as they navigate difficult circumstances to make the best decision for their family. As with any medical decision, we cannot let our personal views impact the conversations we have with patients. We are taught to have equipoise and are held to high professional and ethical standards. Choices regarding abortion and delivery resuscitation are ones that should only be made by patients and their physicians. Legislative restrictions on medically appropriate and sometimes necessary treatments are worrisome. Politics, nonmedical professionals, and fear of litigation or retribution should not dictate the counseling and treatments we provide. We must continue to provide evidence-based medical care to families and ask the Department of Health and Human Services to protect healthcare providers from criminal and civil liability on this matter. Global and regional health policies protecting the interest of our patients are also necessary.

We must remain cognizant of state and federal laws to help families seek abortion services or hospice care when desired and minimize delays in such care. We must call on the healthcare system to provide equitable resources to states that offer abortions so they can facilitate care for citizens regardless of where they live and accommodate the influx of out-of-state patients. This means protected work leave for families, insurance coverage for travel and medical expenses, and support for maintaining and protecting an adequate pool of credentialed physicians and advanced practitioners. We must prepare the healthcare system for more medically complex children. To make pediatric subspecialties and palliative care enticing to trainees we can facilitate work-life balance, promote equity and inclusion, and highlight opportunities for professional growth. The healthcare system will need more physicians, nurses, social workers, ancillary staff, hospitals, equipment, and resources. The government must provide the funding requisite and reform payment structures for hospitals, hospice programs, palliative care resources, and home-health care. Families may require social supports to meet the care needs of children with complex chronic medical conditions. We currently do not have the resources to meet these needs.

Advocacy is important as we navigate the impact of these laws and proposed legislation for our patients. Commitment to advocacy should exist in operational ways that leverage our status as physicians to protect women’s rights, allow physicians and families to determine what is in a neonate’s best interest, decrease maternal and neonatal mortality, and provide resources for infants and families. We must stay current on legislative activities and openly discuss the impact of these rulings with our colleagues so that we can continue to provide the highest level of care. We must advocate for our patients, encouraging them to vote and supporting them in making their voices heard. At the local level, we can educate our patients, communities, and policymakers and call on hospitals to speak out against legislation that shifts medical decision-making to the state. At the state level, we can make our views known to state medical boards and increase our knowledge of how board members are appointed. At a federal level, we can participate in professional organizations and unify their lobbying goals.

Advocacy may come at a cost. Hospitals and institutions may not wish to become publicly involved with a morally and theologically charged topic or be prepared for the financial implications of doing so. Individual involvement may lead to disciplinary action or harm academic advancement. There is a risk of legal repercussions if advocacy equates to supporting illegal activities. It is also important to remember families have views on abortion. They may perceive a physician advocating for decisions regarding abortion to be made by a patient and physician, instead of the government, to be reflective of the physician’s personal views of abortion. This could lead to mistrust or a breakdown of the therapeutic relationship. These concerns, though, should not inhibit physicians from advocating for their patients as individual citizens outside of their affiliation with their employers. Even so, it is imperative that our employers and organizations recognize the gravity of this moment and support physicians in our efforts.

## Conclusion

First and foremost, we must do no harm. As neonatologists we have a great privilege: we care for the most vulnerable individuals. We must meet our ethical obligation to act in the best interests of pregnant individuals, fetuses, and neonates without fear of prosecution altering the care we provide. To think these issues will only impact our obstetric and MFM colleagues is erroneous and short-sighted. These rulings infringe upon the medical community’s ability to make determinations about appropriate care and practice according to established medical and ethical principles. Politicization of medical care undermines the physician/patient relationship we hold sacred, abolishes our ability to provide evidence-based medical care in the best interests of patients, and negates both patient autonomy and parental authority. We must advocate for our ability to continue to practice medicine in the full scope of our specialties.
